# Role of CD25hi CD45RA+ CD4 not Treg %T cell in mediating the effect of pyruvate fermentation to acetone on intrahepatic cholangiocarcinoma

**DOI:** 10.1097/MD.0000000000049498

**Published:** 2026-06-26

**Authors:** Junyu Chen, Hong Li

**Affiliations:** a Department of Hepatobiliary and Pancreatic Surgery, Ningbo Medical Center Lihuili Hospital, Ningbo, China.

**Keywords:** gut microbiota, immune cell, intrahepatic cholangiocarcinoma, mediating effect, Mendelian randomization

## Abstract

This study aimed to elucidate the potential correlation between gut microbiota and intrahepatic cholangiocarcinoma (ICC) by investigating their causal relationship, while also exploring the possible role of immune cells as mediators in this association. We first identified gut microbiota based on phylum, class, order, family, and genus level information. Using summary-level data from a Genome-Wide Association Study (GWAS), we performed a 2-sample Mendelian randomization (MR) analysis of ICC and gut microbiota. Furthermore, we used 2-step MR to quantify the proportion of the effect of immune cell-mediated gut microbiota on ICC. MR analysis identified pyruvate fermentation to acetone (PFA) as predicting ICC risk reduction. There was no strong evidence that genetically predicted ICC had an effect on PFA risk. Furthermore, the proportion of genetically predicted PFA mediated by CD25hi CD45RA+ CD4 not Treg %T cell (CCCTT) was 3% (95% CI: 0.93–5.03%). In conclusion, our study established a causal relationship between PFA and ICC. We observed that a minor fraction of this effect was mediated by CCCTT, while the majority of the impact exerted by PFA on ICC remains elusive. However, further investigations are warranted to elucidate the mechanisms underlying the influence of gut microbiota on ICC development.

## 1. Introduction

Intrahepatic cholangiocarcinoma (ICC) is the second most common primary liver cancer, accounting for up to 20% of all hepatic malignancies and 3% of all gastrointestinal malignancies.^[1]^ The incidence of ICC has increased by more than 140% in the past 4 decades.^[2]^ In China and Southeast Asia, ICC is a prevalent subtype characterized by its high incidence rate, aggressive nature, and unfavorable prognosis.^[3]^

The human symbiotic microbial community consists of >100 trillion microorganisms, including bacteria, viruses, fungi, and protozoa. These microorganisms predominantly colonize the surfaces of human epithelia such as the skin, digestive tract, and respiratory system.^[4]^ The gut microbiota represent the predominant constituents of the human microbial community, exhibiting the highest bacterial abundance and diversity in comparison to other body sites within the microbiome.^[5]^ In recent years, the gut microbiota has garnered significant attention in the fields of gastroenterology and hepatology due to its pivotal role in the pathogenesis and progression of liver and gastrointestinal disorders. Due to the intricate interplay between gut microbiota and bile acid enterohepatic circulation, several studies have indicated a potential correlation between ICC and gut microbiota.^[6]^ However, the causal relationship remains unclear.

Metabolites generated by the gut microbiota exert regulatory effects on genetic and epigenetic processes, as well as modulate immune cell metabolism through their interactions with receptors expressed within these cells.^[7]^ The involvement of immune cells is therefore crucial in the pathogenesis and progression of ICC.^[8]^ Hence, it is postulated that certain immune cells may serve as potential intermediaries linking ICC and specific gut microbiota.

Mendelian randomization (MR) represents a promising methodology for inferring causality by utilizing genetic variation as an instrumental variable to estimate the impact of exposure factors on outcomes in observational data. MR can reduce the effects of nonmeasurement errors or confounding factors while avoiding reverse causality through Mendelian inheritance law. Hence, our study aimed to investigate the existence of a causal association between specific gut microbiota and ICC, as well as identify distinct immune cells and assess their degree of mediation in the observed effects.

## 2. Materials and methods

### 2.1. Study design

The data used in our analysis were publicly accessible and received approval from the institutional review committees of the respective studies. Therefore, no further sanctions were needed. All generated results are presented in this article and its supplements.

In this study, we explored the reciprocal causal relationship between gut microbiota and ICC by 2-sample, bidirectional MR. In our study, single nucleotide polymorphisms (SNPs) were defined as instrumental variables (IVs).

To minimize population stratification bias, this MR study included only adults of European ancestry (aged ≥ 18 years). Eligible participants met 3 criteria: European descent, age ≥ 18 years, and complete data availability. We excluded individuals of non-European ancestry, minors, and those with missing data.

### 2.2. GWAS summary data sources

The summary statistics for the human gut microbiome we used in this study were obtained from a Genome-Wide Association Study (GWAS), which included 207 taxa and 205 pathways. They are available for direct download at the NHGRI-EBI GWAS Catalog under the study accession numbers GCST90027446-GCST90027857.^[9]^ Details of the study have been described elsewhere.^[9]^

Data on ICC were drawn from the GWAS Catalog, which is available at https://www.ebi.ac.uk/gwas/home (ICC including 104 European ancestry cases, 4,56,244 European ancestry controls, accession number GCST90043859).

GWAS summary statistics for each immune trait are publicly available from the GWAS Catalog (accession numbers from GCST0001391 to GCST0002121).^[10]^

A total of 731 immunophenotypes including absolute cell (AC) counts (n = 118), median fluorescence intensities (MFI) reflecting surface antigen levels (n = 389), morphological parameters (MP; n = 32), and relative cell (RC) counts (n = 192) were included. The original GWAS on immune traits was performed using data from 3757 European individuals, and there were no overlapping cohorts.^[11]^

### 2.3. Instrumental variable selection and data harmonization

We included SNPs that were genome-wide significant (*P* < 5 × 10^−8^). If there were no genome-wide significant SNPs as IVs, SNPs with less than a genome-wide significance level (*P* < 5 × 10^−6^) were used as candidate IVs. Then, these SNPs were clustered based on linkage disequilibrium (window size = 10,000 kb and *r*^2^ < 0.001). Estimated levels of linkage disequilibrium were obtained from the 1000 Genomes Project based on European samples. If a particular exposed SNP was not present in the outcome dataset, proxy SNPs were used by LD tagging. Palindromic and ambiguous SNPs were excluded from IVs for Mendelian randomization analysis. The *F*-statistic was calculated by the variance explained by SNPs for each exposure, that is, [(N−K−1)/K]/ [*R*^2^/(1 – *R*^2^)], where K is the number of genetic variants and N is the sample size. We removed weak instrumental variables (*F*-statistics < 10).

### 2.4. Statistical analysis

**Figure 1.**
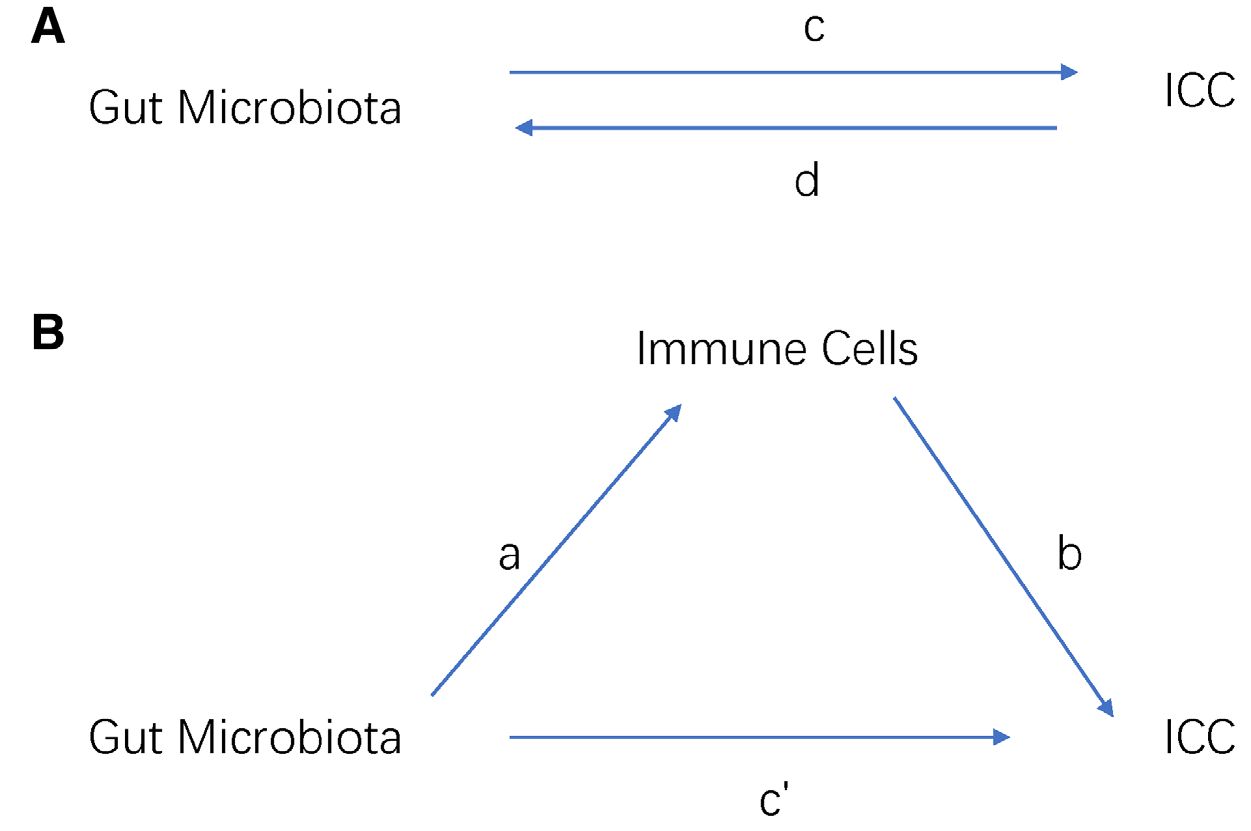
Diagrams illustrating associations examined in this study. (A) The total effect between gut microbiota and ICC. “c” is the total effect using genetically predicted gut microbiota as exposure and ICC as outcome. “d” is the total effect using genetically predicted ICC as exposure and gut microbiota as outcome. (B) Indirect effect using a 2-step approach (where “a” is the total effect of gut microbiota on immune cells, and “b” is the effect of immune cells on ICC), and mediating effects were calculated using the product method (a × b). ICC = intrahepatic cholangiocarcinoma.

We performed MR analysis using R software (version 4.3.2; R Core Team) and the “Two-Sample MR” package (version 0.5.8; MRC-IEU, University of Bristol). Subsequently, Bayesian weighted Mendelian randomization (BWMR) was employed for validation.^[12]^ MR-Pleiotropy RESidual Sum and Outlier (MR-PRESSO) and robust adjusted profile score (MR.RAPS) were performed using the R packages “MRPRESSO” and “MR.raps,” respectively. Calculation of statistical power for Mendelian randomization was performed using mRnd (https://shiny.cnsgenomics.com/mRnd/). We also applied a PhenoScanner search to assess all known phenotypes related to the genetic instruments considered in our analyses. Figure 1 presents a schematic overview of the analysis conducted, wherein we employed a 2-sample bidirectional Mendelian randomization (MR) approach to assess the reciprocal causality between gut microbiota and ICC (Fig. 1A), denoted as the total effect. Inverse variance weighting (IVW) uses meta-analysis to combine the Wald ratios of causal effects for each SNP.^[13]^ Then, MR-Egger and weighted-median methods were used as a complement to IVW.^[14,15]^ Different methods adapted to different validity assumptions were applied to obtain MR estimates. The application of IVW is predicated on the assumption that all SNPs serve as valid instrumental variables. The proposed method, therefore, can yield precise estimation results. In addition, we used Bayesian weighted Mendelian Randomization (BWMR) to verify the reliability of the results.^[12]^

**Figure 2.**
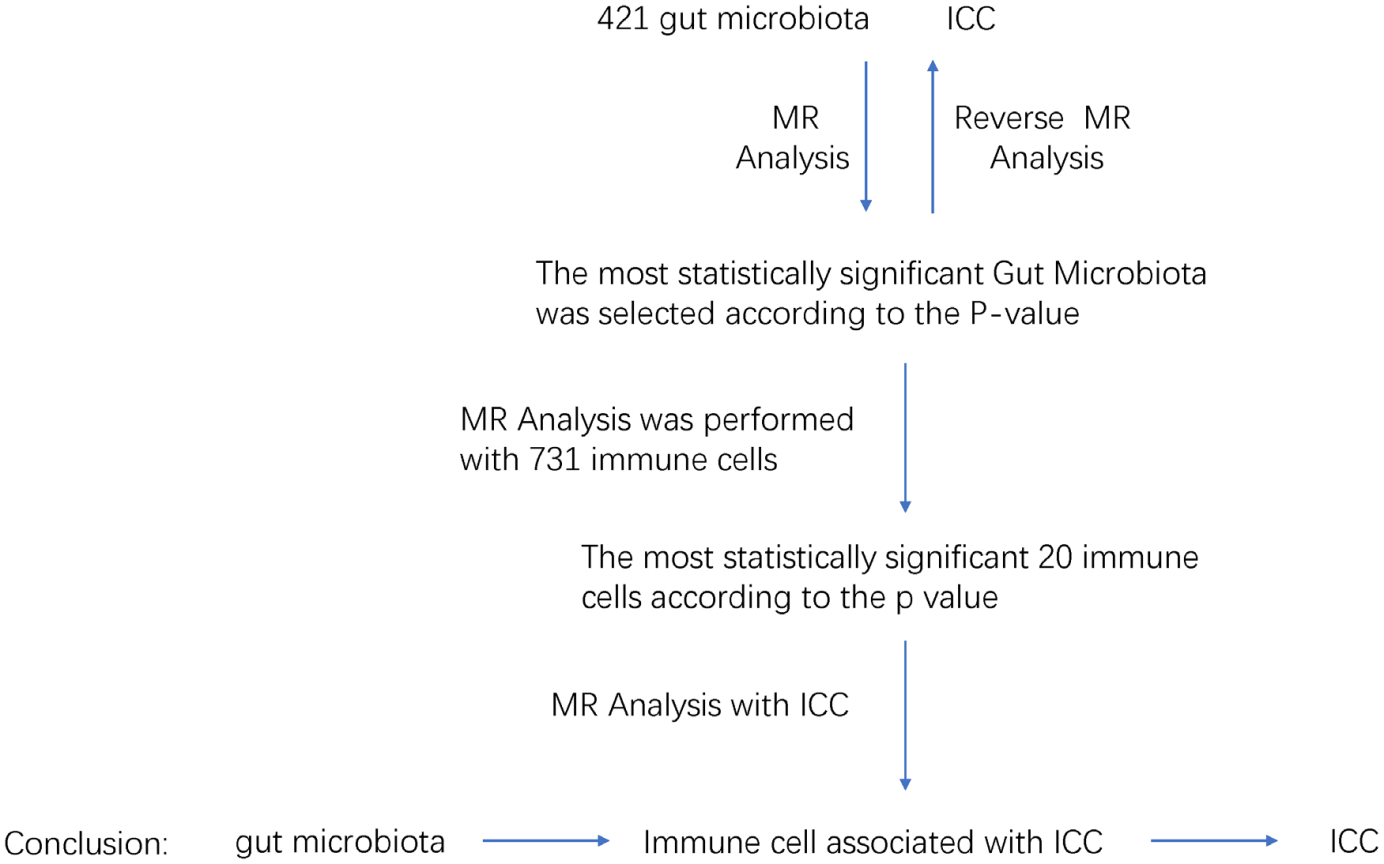
Flow chart. ICC = intrahepatic cholangiocarcinoma, MR = Mendelian randomization.

We further performed a mediation analysis using a 2-step MR design to explore whether Immune Cells mediate the causal pathway from gut microbiota to ICC outcome (Fig. 1B). The overall impact of gut microbiota on ICC was dissected into 2 components: the direct effects exerted by gut microbiota on ICC (c’ in Fig. 1B) and the indirect effects mediated by gut microbiota through the mediator (a*b in Fig. 1B). The flowchart illustrating all processes is presented in Figure 2.

The stability and reliability of the analysis were assessed through sensitivity analysis, heterogeneity tests, pleiotropy tests, and leave-one-out tests. We evaluated the heterogeneity by using Cochran *Q* statistics and *I*^2^ statistics.^[16]^ If heterogeneity existed (*P* < .05), the causal relationship should be consistent with the results estimated by MRE–IVW. In the pleiotropy test, intercepts calculated by MR-Egger regression were used to assess the horizontal pleiotropy of valid IVs. The greater the deviation of the intercepts from zero, the higher the likelihood of horizontal pleiotropy existence (*P* < .05).^[17,18]^

## 3. Results

### 3.1. Main results of the 421 gut microbiota traits with the risk of ICC

**Figure 3.**
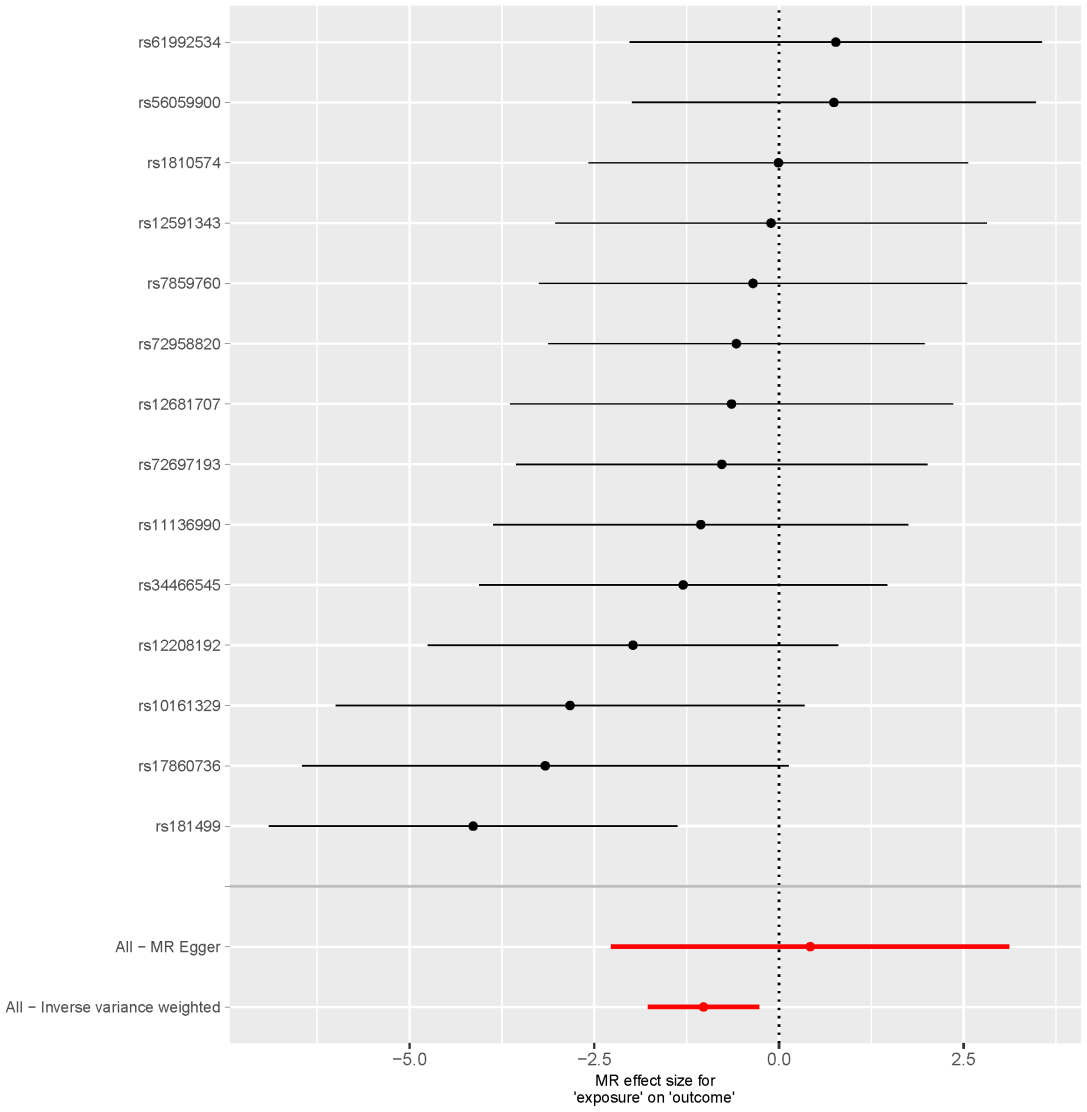
Forest plot to visualize the causal effect of each single SNP on total ICC risk. ICC = intrahepatic cholangiocarcinoma, MR = Mendelian randomization, SNP = single nucleotide polymorphism.

We conducted a comprehensive MR analysis of 421 gut microbiota and ICC, revealing suggestive evidence (*P* < .05) supporting the association between 10 bacterial traits and ICC risk using the IVW method (shown in Supplementary Material 1, Supplemental Digital Content 1). We identified the gut microbiota with the highest statistical significance based on their *P*-values. The pyruvate fermentation to acetone (PFA) was causally associated with ICC (OR = 0.36, 95% CI: 0.17–0.77, *P* = .008). The *F*-statistic of PFA was 20.6, which was above 10, suggesting less possibility of weak instrument bias. Sensitivity analysis showed that there was no pleiotropy or heterogeneity between them (*P* > .05). However, the results of our MR analysis showed no reverse causality for genetically predicted PFA on ICC. Furthermore, Bayesian weighted Mendelian randomization was employed to validate the robustness of our findings (OR = 0.34, 95% CI: 0.15–0.78, *P* = .011). The causal impact of each SNP on the overall risk of ICC is visually represented in Figure 3 through a forest plot.

### 3.2. Association of PFA with 721 immune cells

**Figure 4.**
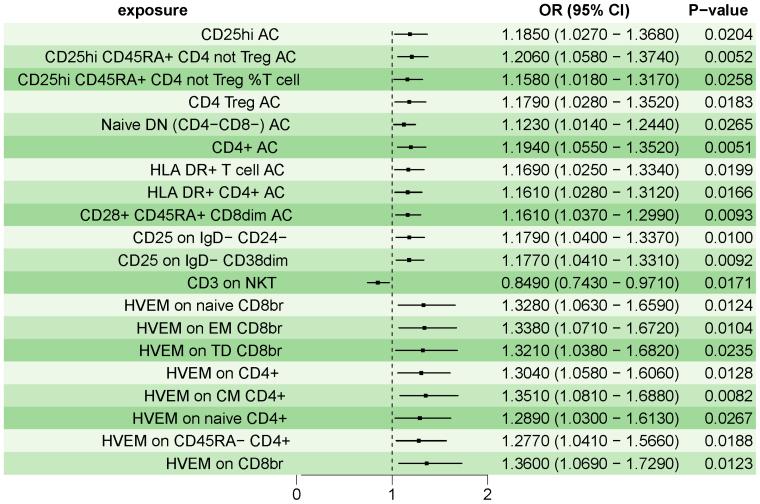
The 20 immune cells with the highest statistical significance were selected based on their *P*-values, and then the forest plot was drawn according to the OR. CI = confidence interval, OR = odds ratio.

We conducted MR analysis on 721 immune cells and investigated PFA, revealing suggestive evidence (*P* < .05) indicating an association between 43 immune cells and the risk of ICC using the IVW method. The 43 suggestive immunophenotypes encompassed 4 in the B cell panel, 14 in the maturation stages of T cell panel, 12 in the TBNK panel, and 13 in the Treg panel (shown in Supplementary Material 2, Supplemental Digital Content 2). We selected the top 20 immune cells with the highest statistical significance, based on their *P*-values, for further analysis. Subsequently, we depicted their odds ratio (OR) values using forest plots (Fig. 4).

### 3.3. Association of immune cells with ICC

We selected 20 immune cells and ICC with the highest statistical significance for MR analysis, based on their respective *P*-values. Genetically predicted CD25hi CD45RA+ CD4 not Treg %T cell (CCCTT) was significantly positively correlated with ICC (OR = 1.23, 95% CI: 1.03–1.46, *P* = .022) by using the IVW method. The statistical significance of MR-Egger and weighted median methods was found to be not significant. Sensitivity analysis revealed no evidence of pleiotropy or heterogeneity between the 2 methods (*P* > .05). Furthermore, BWMR was employed to validate the robustness of our findings (OR = 1.26, 95% CI: 1.03–1.53, *P* = .022).

### 3.4. Proportion of the association between PFA and ICC mediated by CCCTT

**Figure 5.**
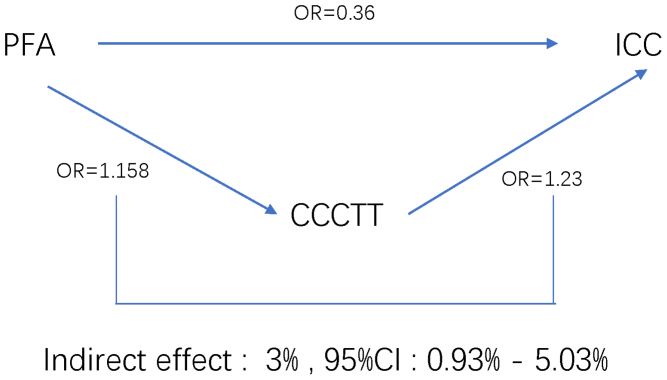
Schematic diagram of the CCCTT mediation effect. CCCTT = CD25hi CD45RA+ CD4 not Treg %T cell, CI = confidence interval, ICC = intrahepatic cholangiocarcinoma, OR = odds ratio, PFA = pyruvate fermentation to acetone.

We investigated CCCTT as a mediator of the pathway from PFA to ICC. We observed a positive correlation between PFA and an elevated proportion of CCCTT, which was found to be associated with an increased susceptibility to ICC. However, we also found PFA was associated with a decreased risk of ICC (all results shown in Table 1); this result may be due to the existence of other mediating factors yet to be found. As shown in Figure 5, our study showed that CCCTT accounted for 3% of the increased risk of ICC associated with PFA (proportion mediated: 3%, 95% CI: 0.93–5.03%).

**Table 1:** The causal effects of CCCTT with PFA and ICC.

Exposure	MR method	OR	OR_lci95	OR_uci95	*P*-value
PFA on ICC	MR-Egger	1.53	0.1	22.61	.764
	Weighted median regression	0.53	0.19	1.52	.2403
	IVW	0.36	0.17	0.77	.008
ICC on PFA	MR-Egger	1.003	0.97	1.039	.82
	Weighted median regression	1.008	0.99	1.024	.27
	IVW	1.001	0.99	1.011	.84
PFA on CCCTT	MR-Egger	1.05	0.69	1.6	.84
	Weighted median regression	1.19	0.99	1.42	.059
	IVW	1.158	1.018	1.317	.0258
CCCTT on ICC	MR-Egger	1.23	0.99	1.52	.07
	Weighted median regression	1.18	0.95	1.47	.13
	IVW	1.23	1.03	1.46	.022

CCCTT = CD25hi CD45RA+ CD4 not Treg %T cell, CI = confidence interval, ICC = intrahepatic cholangiocarcinoma, IVW = inverse variance weighting, MR = Mendelian randomization, OR = odds ratio, PFA = pyruvate fermentation to acetone.

## 4. Discussion

The gut-liver axis, which refers to the connection between the gastrointestinal tract and the liver, plays a pivotal role in regulating not only hepatic diseases but also immune responses within the liver and throughout the organism.^[19]^ As such, the gut microbiome plays a crucial role in modulating antitumor immunity.^[20,21]^ The intestinal barrier serves as the primary line of defense, effectively segregating luminal microbes from the host.^[22]^ Impaired intestinal barrier function and dysbiosis of the gut microbiota have indeed been demonstrated to promote the translocation of gut bacteria to the biliary tract, thereby contributing to the perpetuation of bile duct inflammation. Recent research has investigated the correlation between gut microbiota and ICC.^[6,22,23]^ However, these results may have been influenced by confounding factors. The objective of our study was to elucidate the causal relationship between gut microbiota and ICC. We employed MR analysis to investigate the association between 421 gut microbiota and ICC, based on existing GWAS, aiming to elucidate whether the observed causal relationship is mediated through immune cells. Our findings suggest that there is an inverse association between genetically predicted PFA and the risk of ICC, with approximately 3% of this effect being mediated through CCCTT.

In a recent study, Jia et al^[1]^ discovered an increased abundance of 4 genera (*Lactobacillus*, *Actinomyces*, *Peptostreptococcaceae*, and *Alloscardovia*) in the flora of patients with ICC compared to those with hepatocellular carcinoma (HCC), cirrhosis, and healthy individuals. The combination of *Lactobacillus* and *Alloscardovia* genera enabled discrimination between ICC and the other 3 investigated conditions. And some studies^[24,25]^ have previously shown that primary sclerosing cholangitis (PSC) results in gut dysbiosis in patients. It has been proposed that alterations in the composition of bile acids observed in PSC may influence the gut microbiota.^[24]^ According to our current understanding, PSC is recognized as a significant etiological factor contributing to the development of ICC. Therefore, we hypothesized that specific gut microbiota may exert a significant influence on the pathogenesis and progression of ICC. MR analysis was employed to identify this particular strain of bacteria, while minimizing the inherent bias introduced during the process. These findings suggest that PFA serves as a significant protective factor for ICC.

However, the impact of PFA on the ICC process remains ambiguous. According to a recent study, gut microbiota have been implicated in the regulation of host physiological and immune functions, as well as in the pathogenesis of various diseases through modulation of the immune system.^[26]^ Furthermore, several studies have indicated that the majority of diseases mediated by altered gut microbiota are associated with compromised immune responses.^[27]^ Metabolites derived from the gut microbiota not only exert regulatory effects on genetic and epigenetic processes, but also modulate immune cell metabolism through their interactions with receptors expressed on these cells.^[28,29]^ Zhang et al discovered that gram-negative bacteria and lipopolysaccharide (LPS) induce the accumulation of CXCR2+ polymorphonuclear-MDSCs through TLR4-dependent production of CXCL1, thereby orchestrating an immunosuppressive microenvironment to facilitate cholangiocarcinogenesis in hepatocytes.^[23]^ Therefore, we hypothesize that PFA may affect the generation of ICC through specific populations of immune cells. Utilizing a 2-step MR analysis, we identified CCCTT as the mediating factor responsible for ICC induced by PFA from 731 immune cells. However, it is important to note that the aforementioned outcome solely stems from MR analysis. Therefore, additional experimental investigations are imperative in order to ascertain the reliability of this finding.

The present study has several limitations. First, our analysis was performed using the European population, which limits its prevalence. Second, it is imperative to conduct additional experiments in order to validate the reliability of this outcome, as the current findings solely stem from MR analysis. Third, the results of our study demonstrated a positive correlation between PFA and CCCTT, with an observed increase in the prevalence of ICC associated with elevated levels of CCCTT. However, we also found PFA was associated with a decreased risk of ICC (shown in Fig. 5), which may be due to the existence of other mediating factors that are yet to be identified. Fourth, the mediating factor demonstrated in our study is merely 3%, indicating a significantly low magnitude of influence. Thus, further studies are required to quantify other mediators.

## 5. Conclusion

In conclusion, our study established a causal relationship between PFA and ICC, with a minor proportion of the effect mediated by CCCTT. However, the majority of the impact of PFA on ICC remains indeterminate, and further research is needed on additional risk factors as potential mediators.

## Acknowledgments

Summary statistics for the genetic associations with PTA, CCTTT, and ICC were obtained from GWAS.We thank all investigators for sharing the genome-wide summary statistics

## Author contributions

**Conceptualization:** Junyu Chen, Hong Li.

**Data curation:** Junyu Chen.

**Funding acquisition:** Hong Li.

**Investigation:** Junyu Chen.

**Methodology:** Junyu Chen.

**Project administration:** Junyu Chen.

**Writing – original draft:** Junyu Chen.

**Writing – review & editing:** Hong Li.

## Correction

Additional funding information has now been updated online and hence the funding statement has been changed **from** “*The research was financially supported by the Ningbo Public Welfare Science & Technology Major Project under grant number 2021S106*” **to** “*The research was financially supported by the Ningbo Public Welfare Science & Technology Major Project under grant number 2021S106 and additionally supported by the General Surgery Clinical Key Specialty Construction Project of Zhejiang Province under grant number 2023-SZZ.*”




